# An animal-free preclinical drug screening platform based on human precision-cut kidney slices

**DOI:** 10.1186/s13104-023-06303-4

**Published:** 2023-03-20

**Authors:** Henricus A. M. Mutsaers, Michael Schou Jensen, Jean-Claude Kresse, Stine Julie Tingskov, Mia Gebauer Madsen, Rikke Nørregaard

**Affiliations:** 1 Department of Clinical Medicine, Aarhus University, Palle Juul-Jensens Boulevard 99, Aarhus N, 8200 Denmark; 2 Department of Urology, Aarhus University Hospital, Palle Juul-Jensens Boulevard 99, Aarhus N, 8200 Denmark

**Keywords:** Precision-cut kidney slices, Chronic kidney disease, Renal fibrosis, Alternatives, The 3Rs, Reduction, refinement and replacement

## Abstract

**Objective:**

Renal fibrosis is one of the main pathophysiological processes underlying the progression of chronic kidney disease and kidney allograft failure. In the past decades, overwhelming efforts have been undertaken to find druggable targets for the treatment of renal fibrosis, mainly using cell- and animal models. However, the latter often do not adequately reflect human pathogenesis, obtained results differ per strain within a given species, and the models are associated with considerable discomfort for the animals. Therefore, the objective of this study is to implement the 3Rs in renal fibrosis research by establishing an animal-free drug screening platform for renal fibrosis based on human precision-cut kidney slices (PCKS) and by limiting the use of reagents that are associated with significant animal welfare concerns.

**Results:**

Using Western blotting and gene expression arrays, we show that transforming growth factor-β (TGF-β) induced fibrosis in human PCKS. In addition, our results demonstrated that butaprost, SC-19220 and tamoxifen – all putative anti-fibrotic compounds – altered TGF-β-induced pro-fibrotic gene expression in human PCKS. Moreover, we observed that all compounds modulated fairly distinct sets of genes, however they all impacted TGF-β/SMAD signaling. In conclusion, this study revealed that it is feasible to use an animal-free approach to test drug efficacy and elucidate mechanisms of action.

**Supplementary Information:**

The online version contains supplementary material available at 10.1186/s13104-023-06303-4.

## Introduction

Chronic kidney disease (CKD) is a growing cause of global mortality (28.5 million years of life lost in 2017) and the only available treatment options for patients with end-stage renal disease are dialysis and transplantation [1]. Currently, more than 2.5 million people receive renal replacement therapy globally and this number is projected to double to 5.4 million by 2030 [1]. Renal fibrosis is one of the driving forces underlying CKD progression. This pathological process is characterized by excessive formation and deposition of extracellular matrix proteins by activated (myo)fibroblasts resulting in a loss of organ architecture and function [2]. Therefore, overwhelming efforts have been undertaken to find druggable targets for the treatment of renal fibrosis, mainly using various cell- and animal models. However, drug development is greatly hampered by the lack of suitable translational models of renal fibrosis and as a result current therapeutic modalities are ineffective at preventing fibrogenesis. A Google scholar search for “renal fibrosis” and “animal care”, yielded from 2012 to 2014: 1490 publications; 2015–2017: 2190 publications and from 2018 to 2020: 3100 publications. Random sampling of these papers revealed that an average of 20 animals were used per publication. Thus, every 2 years the number of animals used for renal fibrosis research markedly increased, which resulted in the use of more than 62,000 animals in 2018–2020.

The objective of this study is therefore to implement the 3Rs in preclinical fibrosis research by establishing an animal-free drug screening platform for renal fibrosis based on human precision-cut kidney slices (PCKS) and by limiting the use of reagents that are associated with significant animal welfare concerns [3]. We anticipate that the introduction of this relevant human model for fibrosis research will have considerable impact on the reduction and replacement of animal tests in both academia and industry.

## Main text

### Materials and methods

#### Human precision-cut kidney slices

PCKS were prepared from functional (i.e. eGFR > 60 ml/min/1.73 m²) and macroscopically healthy renal cortical tissue obtained from both male and female patients following tumor nephrectomies, as described previously [4]. In short, slices were prepared in ice-cold Krebs-Henseleit buffer containing 25 mM D-glucose, 25 mM NaHCO_3_, 10 mM HEPES and saturated with carbogen (95% O_2_, 5% CO_2_), using the Alabama R&D Tissue Slicer (previously known as the Krumdieck Tissue Slicer). Subsequently, PCKS were cultured in Williams’ medium E with GlutaMAX containing 10 µg/mL ciprofloxacin and 2.7 g/l D-(+)-Glucose solution at 37 °C in an 80% O_2_, 5% CO_2_ atmosphere while gently shaken. Medium was refreshed every 24 h. To preserve tissue viability, ischemia time between clamping of the renal artery and culturing was limited to 1 h. Patient demographics are presented in Table 1. During experiments, the slices were treated for 48 h with 10 ng/ml recombinant human transforming growth factor-β1 (TGF-β; H8541, Sigma-Aldrich) in the absence or presence of butaprost (50 µM; a selective prostaglandin E_2_ [PGE_2_] EP_2_ receptor agonist; 13741, Cayman), SC-19220 (225 µM; a selective PGE_2_ EP_1_ receptor antagonist; S3065, Sigma-Aldrich) or tamoxifen (5 nM; a selective estrogen receptor modulator; T5648, Sigma-Aldrich). Of note, tamoxifen was only present during the final 6 h. We have previously demonstrated the anti-fibrotic activity of these compounds using cell- and animal models as well as human PCKS [5–7].

**Table 1:** Patient demographics (n = 16)

Sex (% male)	50.0
Age (years)	62.4 ± 15.1
BMI	27.7 ± 5.2
eGFR (ml/min/1.73 m^2^)	82.2 ± 9.0
Ischemia time (min)	36.3 ± 16.4

BMI, body mass index; eGFR, estimated glomerular filtration rate. Values are presented as the mean ± standard deviation

#### Gene expression arrays

Total RNA was isolated using a NucleoSpin RNA II mini kit (Macherey Nagel), following the manufacturer’s instructions. RNA concentration was determined by spectrophotometry at 260 nm and then stored at − 80 °C. cDNA was synthesized from 0.5 µg RNA with the RevertAid First Strand synthesis kit (Thermo Scientific). Gene expression was assessed on a qPCR cycler (AriaMx Real-Time PCR system, G8830A, Agilent Technologies) using Human Fibrosis RT^2^ Profiler PCR Arrays (PAHS-120Z, Qiagen), according to the manufacturer’s instructions. This allowed us to profile 84 fibrosis-related genes simultaneously. Results were analyzed and visualized using the web-based tools from the GeneGlobe Data Analysis Center (https://geneglobe.qiagen.com/us/analyze) and ShinyGO 0.77 (http://bioinformatics.sdstate.edu/go/; [8]).

#### Western blotting

Total protein was extracted using RIPA buffer (10 mM Tris-HCl, 150 mM NaCl, 1 mM EDTA, 1% Triton X-100, 0.5% Sodium Deoxycholate, pH 7.4) supplemented with Phosphatase Inhibitor Cocktail 2 and 3 (P5726 and P0044, Sigma-Aldrich), and a Mini Protease Inhibitor Tablet (11836153001, Roche). Subsequently, 2% SDS and DTT were added to the samples, and they were heated for 15 min at 65 °C. Total protein was separated by SDS/PAGE using 12% Criterion TGX Stain-free gels and subsequently blotted onto a nitrocellulose membrane. Afterwards, the membrane was blocked for 1 h with non-fat dry milk in PBS-T. The blot was then incubated overnight at 4 °C with either recombinant anti-fibronectin antibody (FN; ab206928, Abcam; 1:1000) or anti-α smooth muscle actin antibody (αSMA; ab215368, Abcam; 1:1000) – both are bovine serum albumin and azide free. Afterwards, the membrane was washed with PBS-T and incubated with the secondary antibody (recombinant horseradish peroxidase-conjugated anti-rabbit IgG VHH Single Domain; ab191866, Abcam; 1:4000) for 1 h at RT. Protein levels were visualized with ECL-prime detection reagent and normalized to total protein, as measured using stain-free technology [9].

#### Statistics

Statistics were performed with Graphpad Prism 9.4.1 via either One-way ANOVA followed by Uncorrected Fisher’s LSD test or an unpaired t test. Differences between groups were considered to be statistically significant when p < 0.05.

## Results

**Fig. 1:**
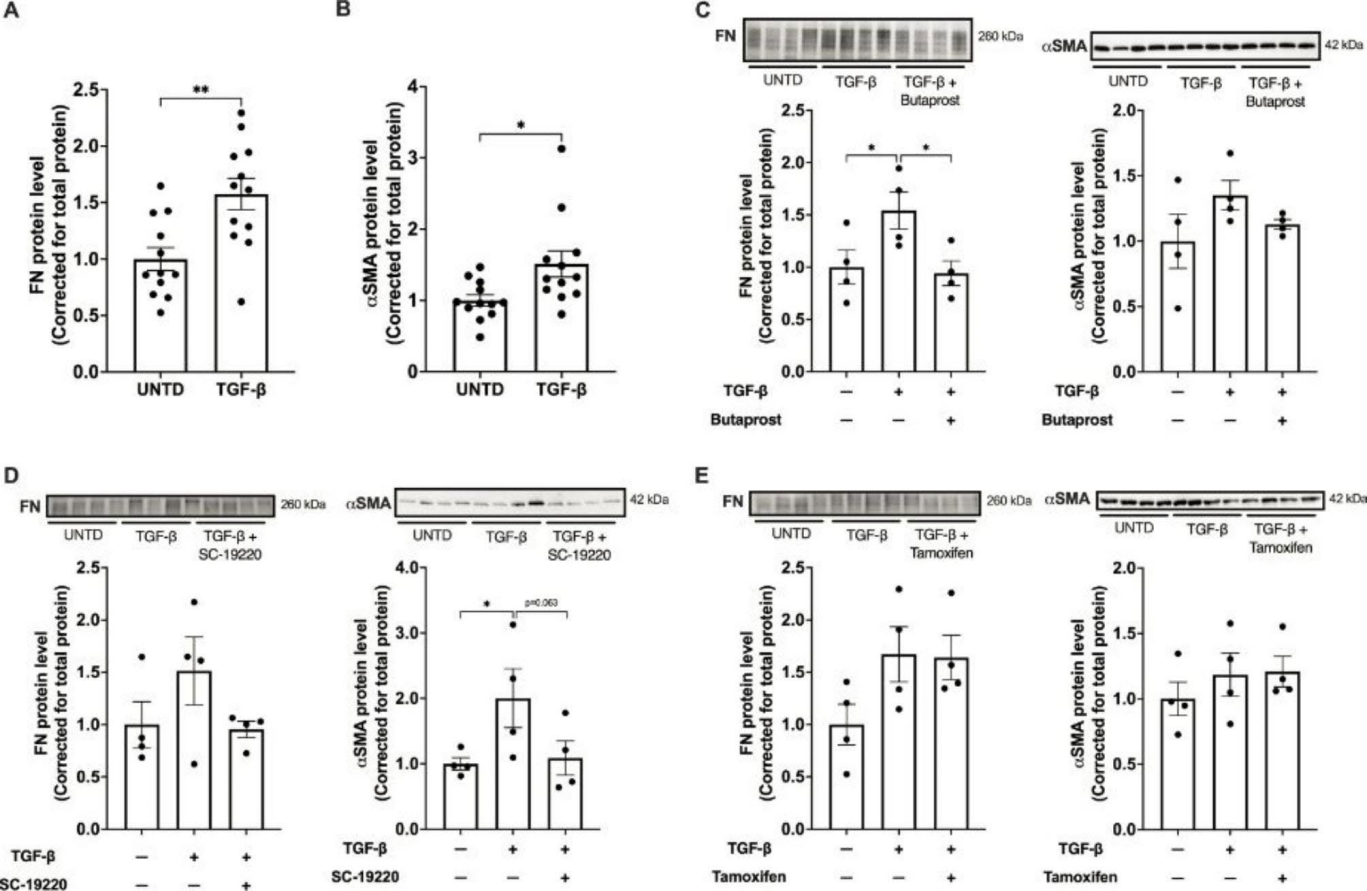
Impact of butaprost, SC-19220 and tamoxifen on TGF-β-induced protein expression in human PCKS. **(A, B)** PCKS were exposed to 10 ng/mL TGF-β for 48 h (n = 12) in the absence or presence of **(C)** butaprost (50 µM), **(D)** SC-19220 (225 µM) or **(E)** tamoxifen (5 nM); n = 4. Of note, tamoxifen was only present during the final 6 h. Subsequently, Western blotting was performed to study the protein expression of fibronectin (FN) and α-smooth muscle actin (αSMA). Data are presented as mean ± SEM. * p < 0.05, ** p < 0.01. Full-length blots are presented in Supplementary Fig. 1. UNTD, untreated

We have previously demonstrated that butaprost, SC-19220 and tamoxifen can attenuate collagen deposition in rodents subjected to unilateral ureteral obstruction [5–7]. Therefore, we now evaluated the impact of all three compounds on the protein expression of FN and αSMA, two well-known markers of activated collagen-producing myofibroblasts [10], in human PCKS. Western blotting revealed that, overall, TGF-β increased the expression of both FN and αSMA (Fig. 1A and B), as expected; however, interindividual differences in response were observed (Fig. 1C-E). In addition, treatment with butaprost and SC-19220 showed a tendency to reduce TGF-β-induced expression of both FN and αSMA (Fig. 1C and D). These results support the notion that prostaglandin E_2_ receptors are potential targets for the treatment of renal fibrosis, in line with previous findings [5, 6, 11]. As shown in Fig. 1E, tamoxifen did not impact TGF-β-induced protein levels, contrary to our previous observations that demonstrated and overall beneficial impact of tamoxifen on TGF-β-induced FN and αSMA gene expression in human PCKS [7].

**Fig. 2:**
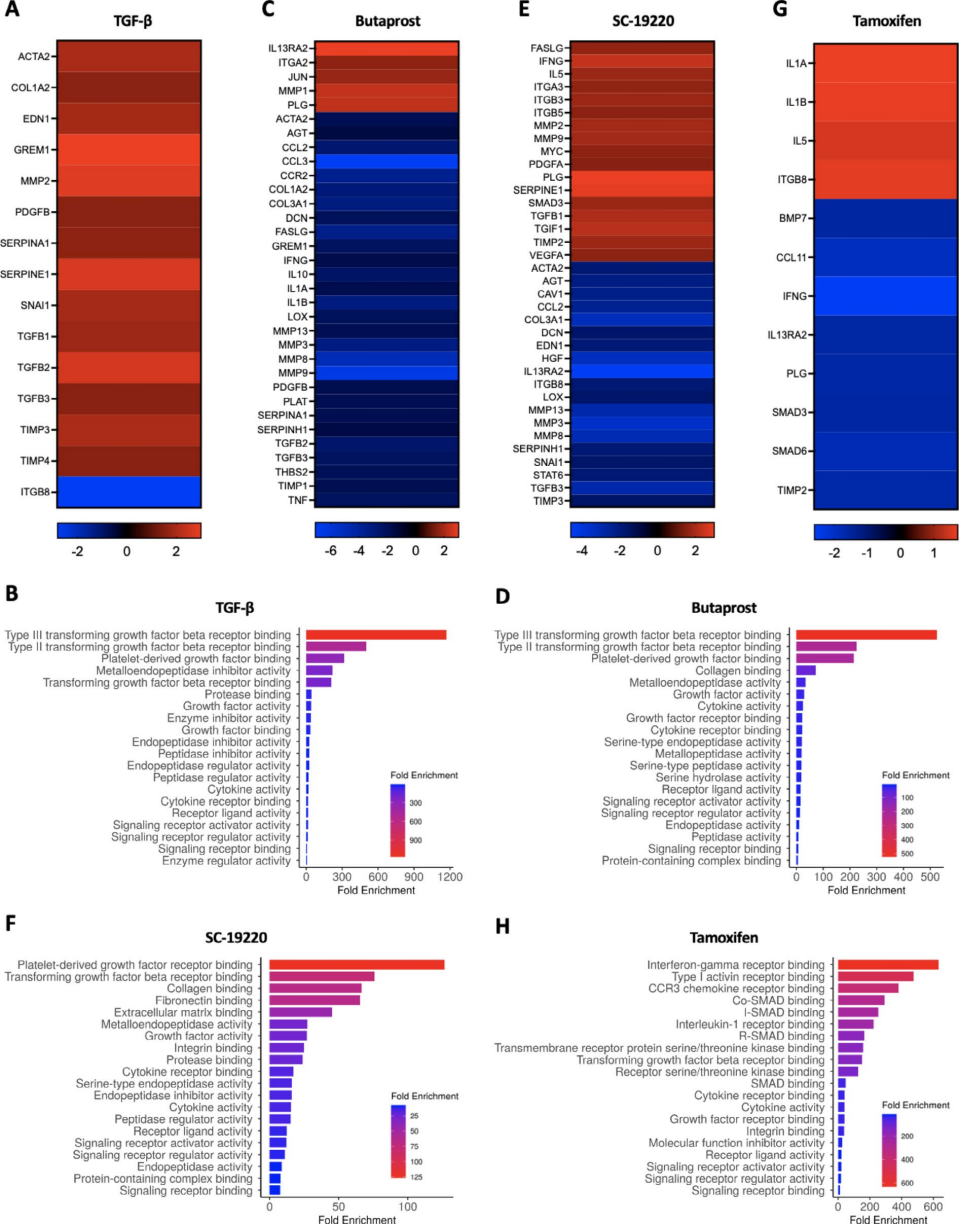
Gene and pathway analysis. Human PCKS were exposed to 10 ng/mL TGF-β for 48 h in the absence or presence of butaprost (50 µM), SC-19220 (225 µM) or tamoxifen (5 nM). Of note, tamoxifen was only present during the final 6 h. Next, gene expression was studied using Human Fibrosis RT^2^ Profiler PCR Arrays. **(A)** Heatmap showing the genes that were regulated more than 1.5-fold following TGF-β exposure. **(B)** Gene ontology analysis for molecular function ranked by fold enrichment. Heatmap showing the genes that were regulated more than 1.5-fold following **(C)** butaprost, **(E)** SC-19220 or **(G)** tamoxifen treatment compared to TGF-β exposed PCKS. Gene ontology analysis for molecular function ranked by fold enrichment for **(D)** butaprost, **(F)** SC-19220 or **(H)** tamoxifen treated slices compared to TGF-β exposed PCKS

Next, we set out to evaluate the potential mechanism of action of all three compounds. Figure 2A shows that treatment with TGF-β resulted in the upregulation (> 1.5-fold) of various pro-fibrotic genes as compared to untreated slices, all of which were strongly associated with TGF-β receptor binding as well as platelet-derived growth factor (PDGF) binding and metalloendopeptidase inhibitor activity (Fig. 2B), as expected. Moreover, we observed that treatment with butaprost or SC-19220 resulted in the downregulation (> 1.5-fold) of multiple genes that strongly correlated with TGF-β receptor binding, PDGF binding and collagen biding (Fig. 2C-F), which is in agreement with previous studies showcasing a close relationship between the PGE_2_ pathway and TGF-β signaling [12, 13]. The genes impacted by tamoxifen treatment were mainly associated with cytokine/chemokine receptor binding and SMAD binding (Fig. 2G and H), in line with previous work [14, 15]. These results demonstrate that the three tested compounds modify the expression of fairly distinct sets of genes but all impact TGF-β/SMAD signaling.

## Conclusion

Our results demonstrated that butaprost, SC-19220 and tamoxifen altered TGF-β-induced pro-fibrotic gene expression in human PCKS, in line with previous studies from our lab [5–7]. Moreover, this study revealed that it is feasible to use an animal-free approach to test drug efficacy and elucidate mechanisms of action.

## Limitations

There are a few limitations to the current study; first off, we only included a small number of patients. However, it has to be taken into account that the present study was an initial proof-of-concept study to demonstrate that it is possible to establish an animal-free drug testing platform that can be used to assess drug efficacy and unveil mechanisms of action. Still, even though our results illustrate and support the applicability of PCKS as animal-free drug screening platform, further optimization and validation is needed before the model can routinely be used in preclinical drug development. Secondly, the arrays we used only contain a relatively small set of predefined genes related to a specific pathological process, namely fibrosis. As such, it is difficult to fully unravel the mechanisms of action of the tested drugs; however, this issue can easily be thwarted by implementing high-powered gene analysis techniques, such as single-cell RNA sequencing (scRNAseq). Studies in which we combine human PCKS and scRNAseq are currently ongoing in our lab. Thirdly, we used non-fat dry milk, an animal product, as blocking agent. Even though, the ethical concerns regarding milk are markedly less severe as compared to other reagents, such as fetal bovine serum, bovine serum albumin or animal-derived antibodies, for a proper implementation of the 3Rs, we do advise the use of commercially available blocking agents that are completely free of animal proteins. Lastly, a drawback of the human PCKS model pertains to the fact that not all academic and industrial research labs can acquire human renal tissue directly from the operating room, which is essential to obtain viable slices. Consequently, it will be difficult for human PCKS to become the gold standard model in preclinical renal fibrosis research. Nevertheless, it is widely acknowledged that the clinical development success rates for investigational drugs is low [16], which is, at least in part, due to the fact that animal experiments often fail to replicate when tested in rigorous human trials [17]. Therefore, it should remain a top priority of the scientific community to develop and implement relevant preclinical models of renal fibrosis with a high predictive value, such as human PCKS [18, 19].

## Electronic supplementary material

Below is the link to the electronic supplementary material.

Supplementary Material 1

## Data Availability

The datasets used and/or analyzed during the current study are available from the corresponding author on reasonable request.

## List of Abbreviations

BMI: Body mass index
CKD: Chronic kidney disease
eGFR: Estimated glomerular filtration rate
FN: Fibronectin
PCKS: Precision-cut kidney slices
PGE_2_: Prostaglandin E_2_
scRNAseq: Single-cell RNA sequencing
TGF-β: Transforming growth factor-β
UNTD: Untreated
αSMA: α-Smooth muscle actin
