# Supplementary material for: An animal-free preclinical drug screening platform based on human precision-cut kidney slices
Source: BMC Res Notes. 2023 Mar 20;16:39. doi: 10.1186/s13104-023-06303-4 (PMC10029185; doi:10.1186/s13104-023-06303-4)

An animal-free preclinical drug screening platform based on human precision-cut kidney slices

Henricus A.M. Mutsaers[1,#,*], Michael Schou Jensen[1,#], Jean-Claude Kresse[1], Stine Julie Tingskov[1], Mia Gebauer Madsen[2] and Rikke Nørregaard[1]

[1]Department of Clinical Medicine, Aarhus University, Aarhus, Denmark
[2]Department of Urology, Aarhus University Hospital, Aarhus, Denmark

Figure 2C Butaprost – FN and αSMA

FN

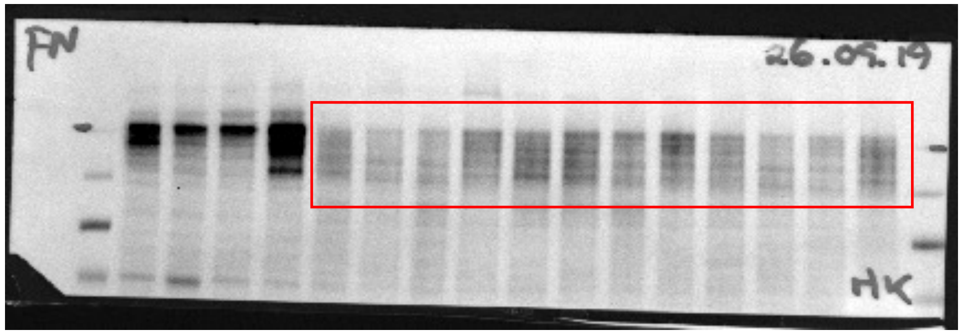

αSMA

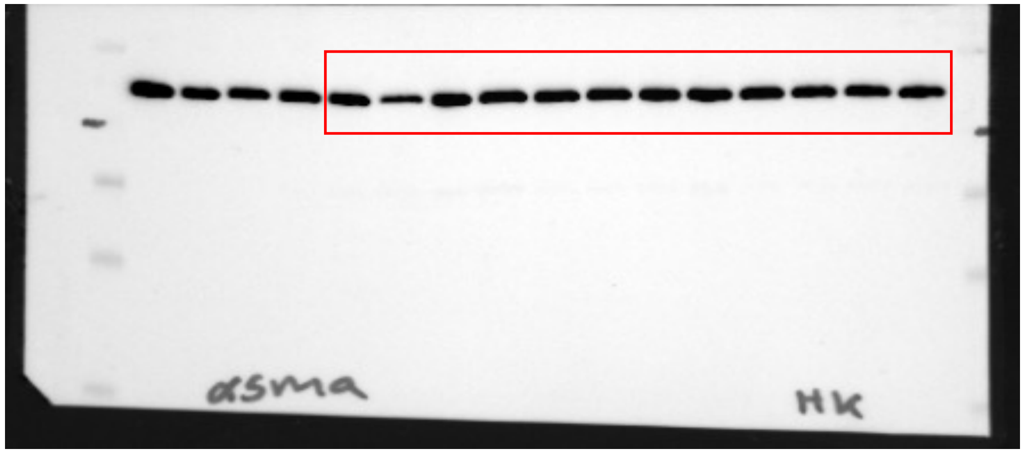

Figure 2D SC-19220 – FN and αSMA

FN

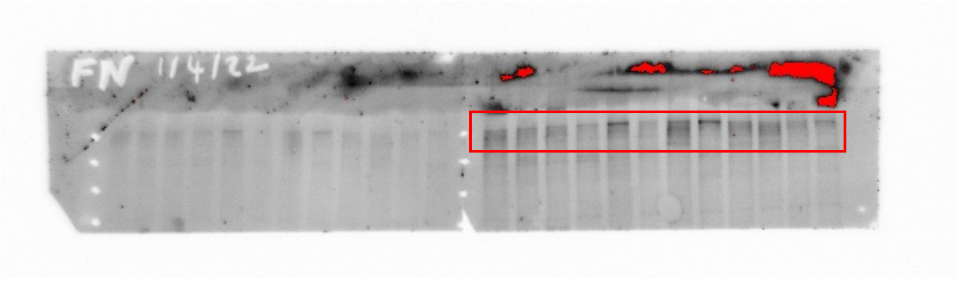

αSMA

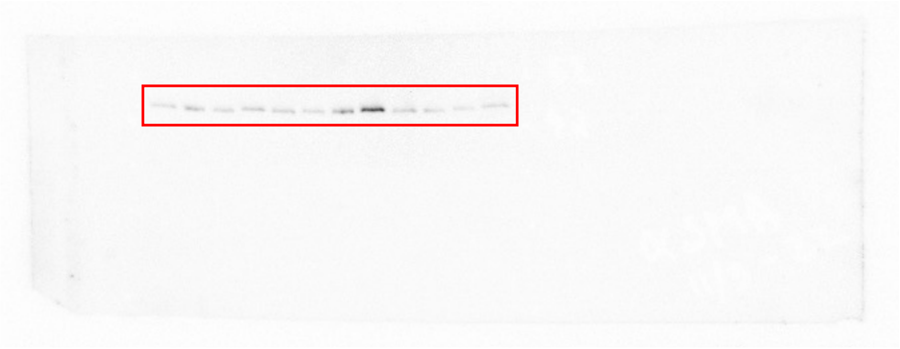

Figure 2E Tamoxifen – FN and α SMA

FN

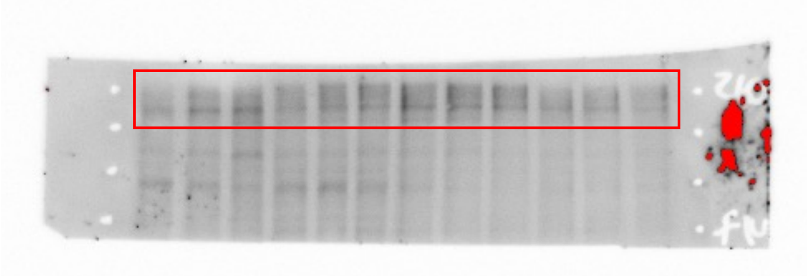

αSMA

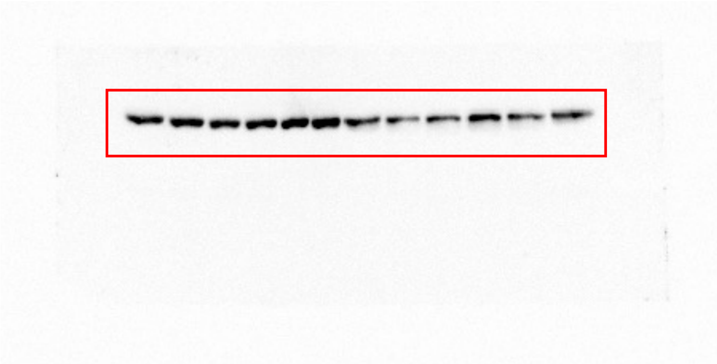

Supplement: Supplementary file 1 — Supplementary Material 1 [file 13104_2023_6303_MOESM1_ESM.pdf]
